# Effect of Adding Y Elements on Microstructure Refinement and Mechanical Properties of Mg-9Al-1Zn Alloy

**DOI:** 10.3390/ma18020259

**Published:** 2025-01-09

**Authors:** Jinhua Cao, Shuaihu Wei, Xiaoming Wang, Xuejian Li, Muyuan Li, Hailong Shi, Xiaoshi Hu, Xiaojun Wang

**Affiliations:** 1 National Key Laboratory of Precision Hot Processing of Metal, Harbin Institute of Technology, Harbin 150001, China; jinhua1589@163.com (J.C.); lixuejian@hit.edu.cn (X.L.); 24b309016@stu.hit.edu.cn (M.L.); hailongshi@hit.edu.cn (H.S.); huxiaoshi@hit.edu.cn (X.H.); 2 Zhengzhou Research Institute, Harbin Institute of Technology, Zhengzhou 450018, China; 3 AECC Harbin Dong’an Engine Co., Ltd., Harbin 150001, China; wxm7256@163.com

**Keywords:** Mg-9Al-1Zn alloy, Al_2_Y phase, grain refinement, mechanical properties, microstructures, Mg_17_Al_12_ phase

## Abstract

Grain refinement is of very important significance for improving the microstructure and mechanical properties of Mg alloys. This work investigated how the addition of various amounts of Y element affected the microstructure and mechanical properties of Mg-9Al-1Zn alloy. It was found that the grain size of Mg-9Al-1Zn alloy decreases as Y element content increases. When 1.2 wt.% Y element content was added, the grain size of Mg-9Al-1Zn alloy could be reduced from 334.2 μm to 134.6 μm, and grain refinement efficiency of 60% was achieved. HRTEM analysis demonstrated a good orientation relationship between the Al_2_Y phase and the Mg interface, and therefore, it was determined that the Al_2_Y phase can be used as a heterogeneous nucleation site for α-Mg grains, which promotes the grain refinement of α-Mg grain. Furthermore, the addition of Y element can also significantly refine the eutectic Mg_17_Al_12_ phase of the Mg-9Al-1Zn alloy. In this way, the mechanical properties of the Mg-9Al-1Zn alloy are significantly enhanced. It was found that the YS and UTS of the Mg-9Al-1Zn alloy reached 87 MPa and 154 MPa with the addition of 0.9 wt.% of Y element—24% and 43% higher than the Mg-9Al-1Zn alloy.

## 1. Introduction

Magnesium (Mg) alloys have been extensively used in aerospace applications and in the creation of cars, as well as in the development of new energy sources and electronic digital applications, owing to their light density, higher specific stiffness and strength, and excellent electromagnetic shielding properties [1,2,3]. The casting process is currently the main method used for Mg alloy molding, especially in the industrial setting. Large-volume equipment is created using casting molding methods; this includes aircraft engine shells, automobile wheels, and so on [4]. Mg-Al series alloys, represented by casting Mg alloy, are in widespread use because of their outstanding mechanical properties and good molding performance; thus, they are the most widely used Mg alloys at present [5,6]. However, in the process of Mg-Al series alloy casting molding, due to the relatively wide liquid phase interval, the segregation of Al elements, and the rapid growth of the phase, the microstructure of the casting products tends to have coarse grains, poor mechanical properties, and other problems, seriously limiting the products’ application and development [7,8]. Therefore, research within the field of Mg alloys has begun to focus on regulating the microstructure of Mg-Al series alloys during the casting process and improving their mechanical properties. 

It is well known that grain refinement has a very positive effect on the microstructure of alloys, enhancing their mechanical properties [9,10,11]. In particular, for Mg alloys with a hexagonal crystal structure, the grain refinement effect is more significant, as they have a larger Hall–Peach coefficient (K) compared to aluminum and zinc alloys [12]. Therefore, in recent years, a great deal of research has been carried out with regard to the refinement of the grain structure of Mg alloys, mainly encompassing the external field treatment method, the melt inoculation method, and the plastic deformation method [13,14,15]. Compared with other methods, the melt inoculation method acts directly on the Mg alloy melt, offering significant advantages such as simplicity, high efficiency, and ease of operation, and thus is widely used in the industrial field. Although the addition of Zr-containing modifiers has become the main choice of melt treatment method for magnesium alloys, grain refinement for the widely used Mg-Al series alloys has been problematic because the Zr element tends to react with the Al element and thus loses its refining effect [16]. The development of highly efficient and stable grain refiners for Mg-Al alloys has been an active field.

Over the last few years, studies investigating grain refiners for Mg-Al series alloys have mainly included three types of refiner: carbon-containing modifiers, heterogeneous particles, and alloying elements [17,18]. For example, Chen et al. [19] added 1.2 wt.% content of MgCO_3_ particles to AM60 alloy and observed a reduction in the average grain size of the AM60 alloy from 348 µm to 69 µm. Qiu et al. [20] added 0.5 wt.% of VN particles to the AZ31 alloy and observed that it was possible to reduce the grain size of the AZ31 alloy from 115.7 μm to 62.4 μm. However, either the addition of carbon-containing modifiers or heterogeneous particles is incorporated into the Mg-Al alloy melt by additive processes. These methods are susceptible to the wettability of the Mg/C interface and heterogeneous particle size and distribution, ultimately making the grain refinement unsatisfactory. In comparison, directly adding alloying elements to the melt utilizes the diffusion motion of solute atoms, which results in an excellent grain refinement effect [21,22]. This method is widely used for its high efficiency, convenient operation, and stable grain refinement effect. For example, Qiu et al. [23] used E2EM model calculations to determine that Y element addition can achieve grain refinement of Mg-Al alloys and found that when 0.6~1.5 wt.% content of Al element is added to Mg-10Y alloys, it can make the alloy’s grain size reduce from 180 μm to 36 μm. In addition, Wang et al. [24] added 1.5 wt.% of Al element to the Mg-4Y-3Nd alloy and found that the addition of Al significantly reduced the grain size and improved the thermal stability of the alloy. Some scholars have investigated the effect of Y element addition on the grain structure refinement of Mg-Al alloys. However, the effect of Y element addition on the morphology and structure of the eutectic Mg_17_Al_12_ phase of Mg-Al alloys has not been studied in detail. In addition, the specific mechanism of the Y element on the microstructure evolution of Mg-Al series alloys is still unclear, and the effect on the mechanical properties of Mg-Al alloys also remains unclear.

Hence, in this work, we have conducted a detailed investigation of the mechanism of the effect of Y element addition on the grain refinement and mechanical properties (e.g., yield strength, ultimate tensile strength, and elongation) of Mg-9Al-1Zn alloys. The influence of Y element addition on the second-phase morphology and distribution in the Mg-9Al-1Zn alloy was analyzed by OM, SEM and TEM. Further, the interface relationship of Al_2_Y/Mg was clearly identified using a high-resolution transmission electron microscope (HRTEM).

## 2. Materials and Experimental Procedure

### 2.1. Materials

Pure Mg, Al and Zn were used as raw materials; these were obtained from Zhongke Metals Research Institute. These materials were melted within a resistance furnace in a protective atmosphere of pure SF_6_ and CO_2_ gas and held at 720 °C for 20 min. The matrix alloy Mg-9Al-1Zn required for the experiment was obtained. Subsequently, various amounts of Y element were mixed into the pure Mg alloy, which was accompanied by manual stirring for 30 s and holding for 10 min. Lastly, the melt was placed in a stainless-steel mold to obtain the ingot (Mg-9Al-1Zn-xY; x = 0, 0.3 wt.%, 0.6 wt.%, 0.9 wt.%, 1.2 wt.%).

### 2.2. Microstructural Characterization

The microstructures of the alloy were analyzed by optical microscopy (OM), scanning electron microscopy (SEM, Helios 5 CX, Thermo Fisher Scientific, Waltham, MA, USA), transmission electron microscopy (TEM), etc. Due to the differing electrochemical potentials of the matrix Mg and the eutectic Mg_17_Al_12_ phases, which led to different corrosion rates, it was possible to identify the grain structure of the Mg-9Al-1Zn alloy by chemical corrosion. The samples used for OM observation were polished and etched with a mixed solution containing 2 g picric acid, 5 mL glacial acetic acid, 2 mL distilled water, and 60 mL ethanol. Tensile tests were carried out with an MST/E45.105 tensile testing machine at a tensile rate of 0.5 mm/min. The interface relationship of Al_2_Y/Mg was characterized by a Talos F200X High-Resolution TEM (HRTEM). The average grain size was calculated by randomly scribing 100 grains from several metallographic images and obtaining a normal distribution data plot to obtain the average value and, finally, the average grain size of the alloy.

## 3. Results and Discussion

**Figure 1:**
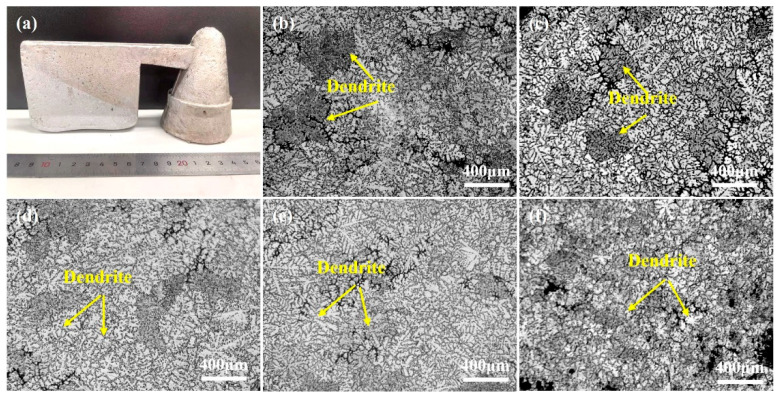
Optical morphology and microstructure of Mg-9Al-1Zn-xY alloy: (**a**) Optical morphology of the Mg-9Al-1Zn-xY alloy ingot; (**b**) Mg-9Al-1Zn alloy; (**c**) Mg-9Al-1Zn-0.3Y alloy; (**d**) Mg-9Al-1Zn-0.6Y alloy; (**e**) Mg-9Al-1Zn-0.9Y alloy; and (**f**) Mg-9Al-1Zn-1.2Y alloy.

Figure 1 shows the optical morphology of the original ingot and the microstructure of the Mg-9Al-1Zn-xY alloy obtained by OM. The original ingot prepared using the metal mold antigravity casting process is shown in Figure 1a. It can be seen that the surface of the casting is smooth and neat with no major void defects, indicating that the casting process is suitable. Figure 1b–f demonstrate the microstructure evolution of Mg-9Al-1Zn alloy when various amounts of Y element are added. It can be seen through Figure 1b that the grain structure of Mg-9Al-1Zn alloy is coarse and irregular dendritic when no Y element is added. With the increase in Y element content, the grain size of Mg-9Al-1Zn alloy is continuously refined, and its coarse dendritic crystals are gradually transformed into a fine and uniform equiaxed structure. This indicates that the Y element can significantly refine the microstructure of Mg-9Al-1Zn alloy.

**Figure 2:**
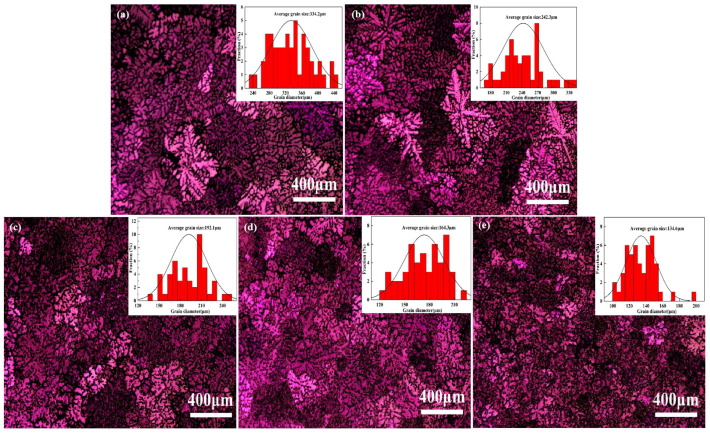
The polarized light microstructure of Mg-9Al-1Zn-xY alloy (the top right inset shows the average grain size statistic of the alloy): (**a**) Mg-9Al-1Zn alloy; (**b**) Mg-9Al-1Zn-0.3Y alloy; (**c**) Mg-9Al-1Zn-0.6Y alloy; (**d**) Mg-9Al-1Zn-0.9Y alloy; and (**e**) Mg-9Al-1Zn-1.2Y alloy.

Figure 2 demonstrates the polarized light metallographic structure of the Mg-9Al-1Zn alloy after the addition of different amounts of Y elements, and the grain size distribution of the alloy is statistically analyzed using Image J software (4.2). From Figure 2a, it can be observed that the Mg-9Al-1Zn alloy without any additional Y element has a large dendritic and a grain size of about 334.2 μm. When the Mg-9Al-1Zn alloy is treated with 0.3 wt% of element Y, its grain is significantly refined and the average grain size reduces to 242.3 μm, as shown in Figure 2b. In addition, the statistical plot of grain size reveals that the peak grain size of the alloy is significantly shifted and the number of large-sized grains is significantly reduced after the addition of Y element compared to the pristine Mg-9Al-1Zn alloy. The average grain size of the Mg-9Al-1Zn alloy is further reduced to 192.1 μm and 164.3 μm in Figure 2c,d as the Y element content increases to 0.6 wt.% and 0.9 wt.%, relatively. When the Y element content is increased to 1.2 wt.%, it can be seen via Figure 2e that the grains of Mg-9Al-1Zn alloy are greatly refined and the structure of the grain becomes finer and more homogeneous, and the average grain size is reduced to 134.6 μm. Meanwhile, the distribution of grain size is mainly concentrated at 100~160 μm. This suggests that adding Y element can significantly help to refine the grain of Mg-9Al-1Zn alloy.

**Figure 3:**
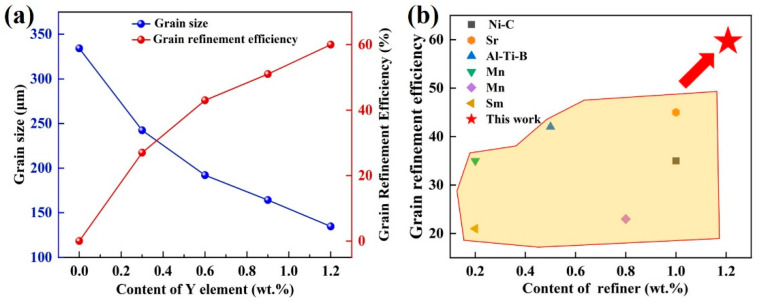
Comparative statistics on the grain size and the refinement performance of Mg-9Al-1Zn-xY alloys: (**a**) Grain data analysis of Mg-9Al-1Zn-xY alloys; (**b**) comparison of refinement efficiency with a variety of Mg alloy grain refiner [25,26,27,28,29,30].

Figure 3 demonstrates the grain data analysis of the Mg-9Al-1Zn alloy following the various Y-element additions, including the average grain and the efficiency with which the grains are refined. As shown in Figure 3a, the statistical analysis indicates that the specific average values for grain size of the Mg-9Al-1Zn alloy continue to decrease as the Y-element level increases, and the grain refinement efficiency increases. This demonstrates element Y’s sustained grain-refining ability for Mg-9Al-1Zn alloys. The average value on the grain of Mg-9Al-1Zn alloy is reduced from 334.2 μm to 134.6 μm when 1.2 wt.% content of Y element is added, and the grain refinement efficiency reaches 60%. By comparing the grain refinement efficiency of Mg alloys with different alloying element refiners, as shown in Figure 3b, we have found that the Y element has a very good modification performance for Al-containing cast Mg alloys [25,26,27,28,29,30].

**Figure 4:**
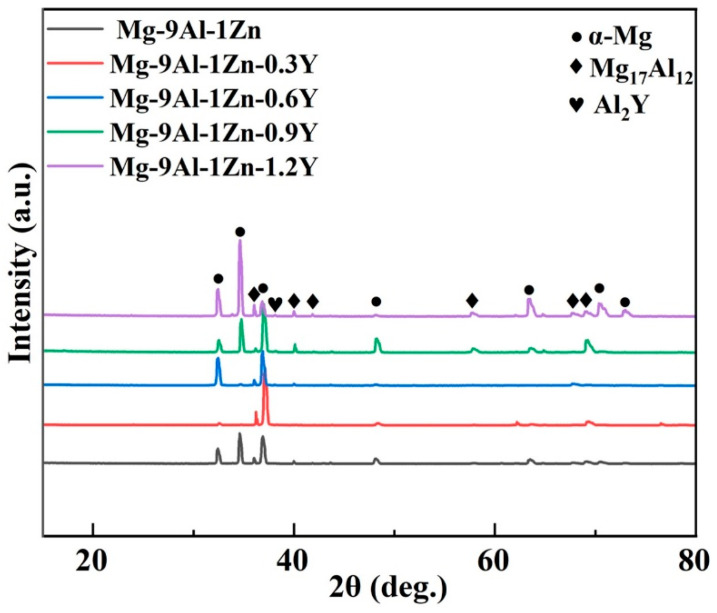
The XRD analysis of Mg-9Al-1Zn-xY alloys.

Figure 4 illustrates the XRD analysis of the Mg-9Al-1Zn alloy with the addition of different amounts of Y element. The Mg-9Al-Zn alloy without any additional Y elements consists mainly of α-Mg and Mg_17_Al_12_ phases, as shown in Figure 4. When Y element is added to the matrix, it can be observed from the XRD pattern that Al_2_Y peaks with different intensities appear at the peak position of 38°, and as the Y element content increases, these peaks increase in height. This demonstrates that the added Y element can achieve in situ reaction with the element of Al in the matrix to form the Al_2_Y phase. Due to the excellent orientation relationship between the Al_2_Y phase and the matrix α-Mg crystals, it can effectively promote the heterogeneous nucleation of the α-Mg grains, therefore contributing to the refinement of the grain. With the increase in Y element content, the Al_2_Y particles in the melt gradually increase, which can promote the increase in particle density of heterogeneous nucleation, and therefore, the grain refinement effect becomes more and more obvious.

**Figure 5:**
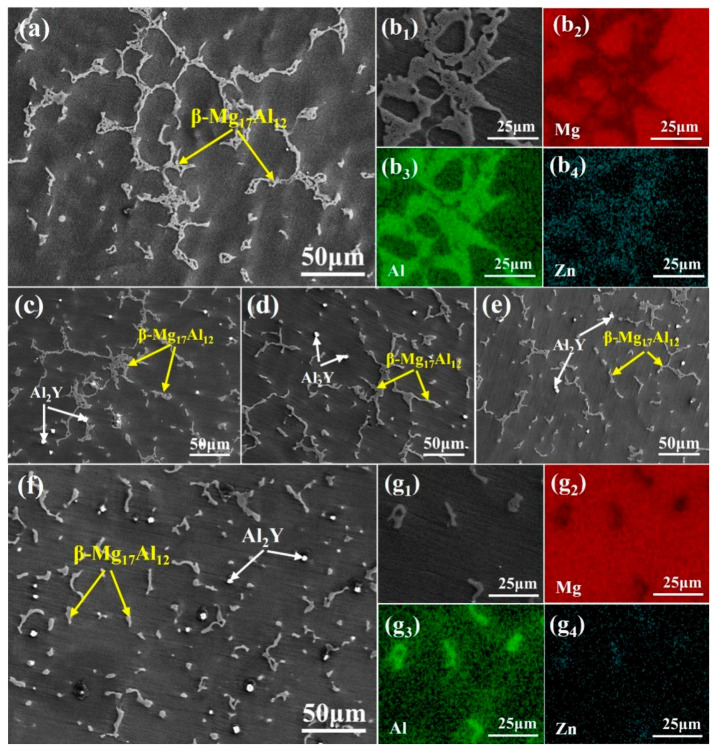
SEM and EDS analysis of the microstructure of Mg-9Al-1Zn-xY alloy: (**a**) Mg-9Al-1Zn alloy; (**b_1_**–**b_4_**) the EDS analysis of the Mg_17_Al_12_ phase in Mg-9Al-1Zn alloy; (**c**) Mg-9Al-1Zn-0.3Y alloy; (**d**) Mg-9Al-1Zn-0.6Y alloy; (**e**) Mg-9Al-1Zn-0.9Y alloy; (**f**) Mg-9Al-1Zn-1.2Y alloy; and (**g_1_**–**g_4_**) the EDS analysis of the Mg_17_Al_12_ phase in Mg-9Al-1Zn-1.2Y alloy.

Figure 5 shows the SEM characterization of the microstructure of the Mg-9Al-1Zn alloy after the addition of different Y-element contents. The continuous striated phase in Figure 5 is the eutectic Mg_17_Al_12_ phase, the gray phase represents the primary phase α-Mg, and the white particle-like phase is the Al_2_Y phase. As shown in Figure 5a, the Mg matrix mainly contains a continuous coarse eutectic Mg_17_Al_12_ without the addition of element Y. The EDS spectral analysis of Figure 5b reveals that the phase contains two major elements, Mg and Al, which verifies that the phase is Mg_17_Al_12_. Further observations reveal that the eutectic Mg_17_Al_12_ phase in the Mg-9Al-1Zn alloy is continuously refined as the Y element level increases, and its morphology gradually changes from the continuous “network structure” into a discontinuous “strip structure”, while a white particle Al_2_Y phase appears inside the α-Mg grains. When 1.2 wt.% Y element content is added, as shown in Figure 5f, it is found that the eutectic Mg_17_Al_12_ phase is significantly refined, and the morphology is completely altered to a discontinuous “point-like structure”. This indicates that the addition of the Y element may be able to significantly refine the grains and can have a considerable influence on the eutectic tissue. The eutectic Mg_17_Al_12_ phase is mainly precipitated and distributed along the α-Mg grain boundaries during solidification. With the addition of Y element, the α-Mg grains are significantly refined, which makes the density of grain boundaries in the melt increase significantly and makes the distribution of eutectic Mg_17_Al_12_ phase more dispersed; therefore, the eutectic Mg_17_Al_12_ phase is significantly refined [31,32].

**Figure 6:**
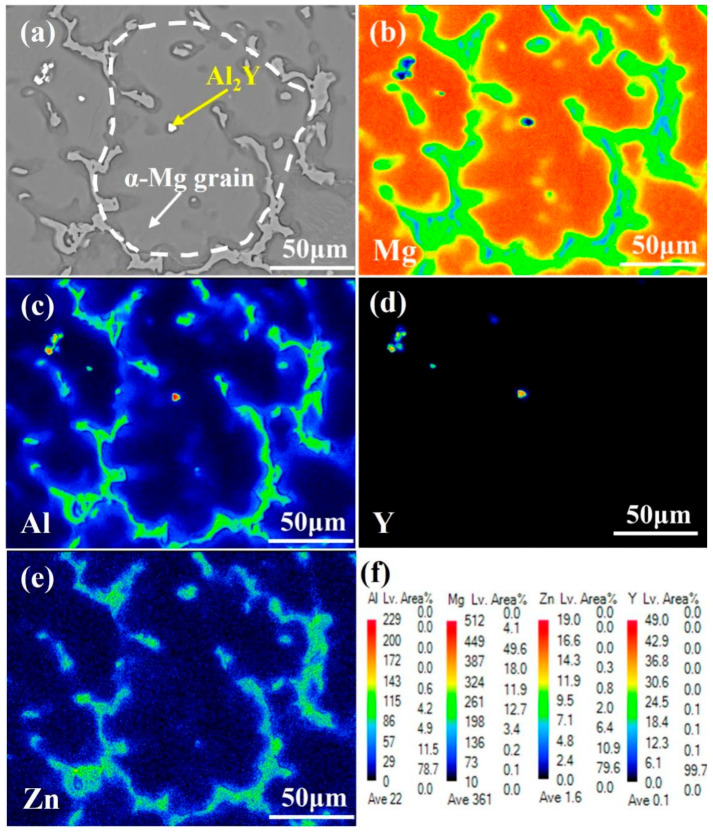
EPMA analysis on the heterogeneous nucleation of α-Mg grains on the surface of the Al_2_Y phase in Mg-9Al-1Zn-1.2Y alloy: (**a**) SEM image on the nucleation of α-Mg grains; (**b**–**e**) the distribution of Mg, Al, Y, and Zn elements in (**a**) analyzed by EPMA; and (**f**) the intensity scale of elemental distribution for EPMA analysis.

Figure 6 exhibits the EPMA analysis of α-Mg grains nucleated at the surface of the Al_2_Y phase in the Mg-9Al-1Zn-1.2Y alloy. As shown in Figure 6a, the presence of a white particle phase in the interior of α-Mg grains is observed using SEM. The phase is analyzed using EPMA and is found to contain mainly Al and Y elements, as shown in Figure 6b–e, proving that the phase is an Al_2_Y phase formed in situ. It is demonstrated that the formation of Al_2_Y phase can effectively serve as a point where nucleation is induced for α-Mg grains in the melt, which promotes the appearance of α-Mg grain nucleation on its surface and ultimately provides a significant grain-refinement effect.

**Figure 7:**
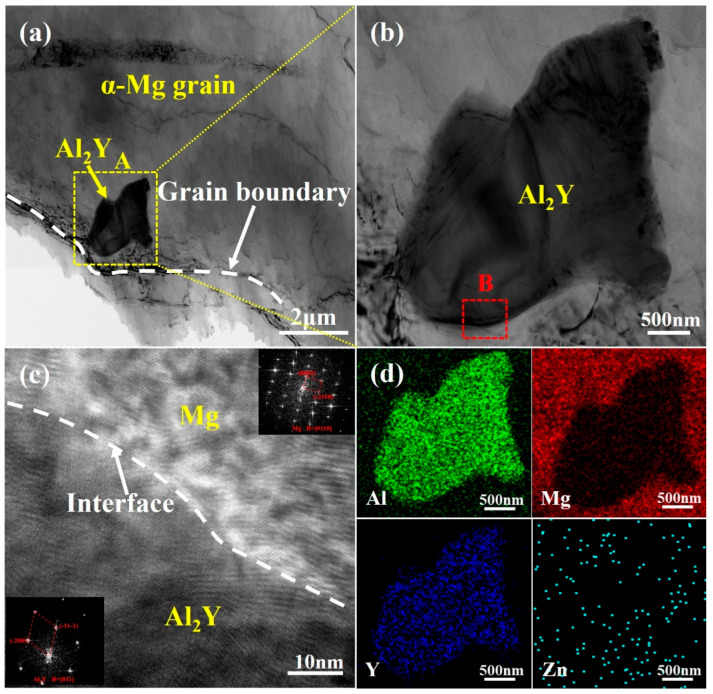
The TEM analysis of the Al_2_Y phase in the Mg-9Al-1Zn-1.2Y alloy: (**a**) TEM morphology of Al_2_Y phase in the interior of α-Mg grains; (**b**) higher magnification of the yellow box in (**a**); (**c**) the HRTEM of the red box in (**b**); and (**d**) EDS energy spectrum analysis of (**b**).

Figure 7 demonstrates the TEM morphology and elemental distribution of the Al_2_Y phase in the Mg-9Al-1Zn-1.2Y alloy. As shown in Figure 7a, the presence of in situ formed Al_2_Y phase inside the α-Mg grains is observed by TEM. The size of the in situ formed Al_2_Y phase is about 2 μm with polygonal morphology, as observed by the high-magnification TEM in Figure 7b. The EDS energy spectrum analysis of Figure 7d illustrates that the particles mainly include elements Al and Y, proving that the phase is an Al_2_Y phase. A good interfacial relationship between the nucleating particle and the matrix is essential to ensure that heterogeneous nucleation can occur [33]. Therefore, the interface between Al_2_Y and Mg is analyzed using HRTEM, as shown in Figure 7c. It can be observed that there is a good interfacial relationship between the Al_2_Y phase and the matrix α-Mg. No defects or voids are found to exist at the interface, and the overall interface is smooth. The inset in Figure 7c on the two-phase diffraction spots further proves that the interface is composed of Al_2_Y and Mg. The conclusion that Al_2_Y can promote heterogeneous nucleation of α-Mg grains has also been demonstrated in previous work. Qiu et al. [23] predicted that the Al_2_Y phase can be a nucleation promoter for α-Mg grains using an E2EM crystallographic model and verified it experimentally. In addition, Wang et al. [24] found that the Al_2_Y phase has a good crystallographic orientation relationship with α-Mg, specifically (011)_Al2Y_//(01-10)_Mg_, with a lattice misfit of only 0.65%. Due to the low lattice mismatch between the Al_2_Y phase and the matrix α-Mg grains, it indicates a low interfacial energy between the two phases, which facilitates the adsorption of α-Mg grains on the surface of the Al_2_Y phase for nucleation. Thus, the above analysis demonstrates that the Al_2_Y phase can act as a heterogeneous particle to facilitate α-Mg de-nucleation and growth, ultimately refining the alloy grains.

**Figure 8:**
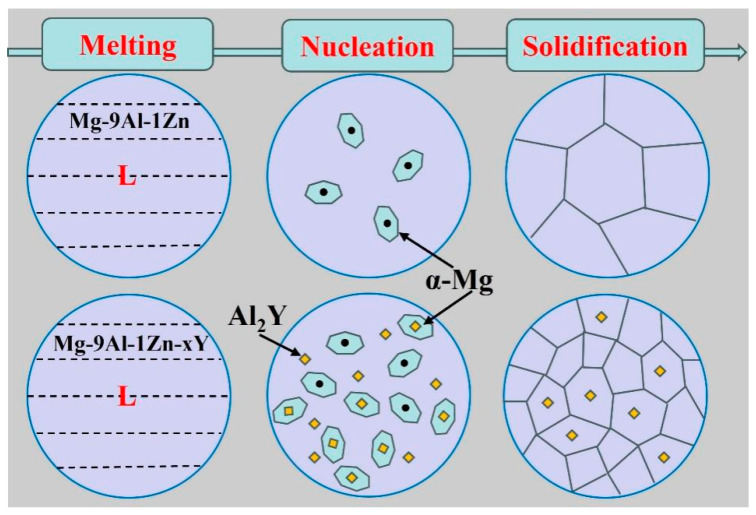
Schematic showing the refinement mechanism of Mg-9Al-1Zn-xY alloys.

Figure 8 exhibits a schematic draft of the mechanism of the effect of the addition of element Y on the grain refinement of the Mg-9Al-1Zn alloy melt. It can be seen through Figure 8 that the α-Mg grains can only rely on the small amount of impurity elements present in the melt to undergo heterogeneous nucleation in the alloy melt without adding the Y element, and thus, due to the small number of effective nucleation sites, the nucleation efficiency of the α-Mg grains is low, resulting in grain coarsening of the Mg-9Al-1Zn alloy. However, when the Y element is added to the Mg-9Al-1Zn alloy melt, the Al element in the matrix can spontaneously react chemically with the added Y element, forming the Al_2_Y phase. Due to the good orientation relationship (OR) between the matrix α-Mg and the spontaneously formed Al_2_Y particles in the crystal structure, it can be used as a site for inducing nucleation of α-Mg grains, which significantly increases the nucleation density of α-Mg grains, enhances the nucleation efficiency, and ultimately leads to a significant grain refinement in the Mg-9Al-1Zn alloy.

**Figure 9:**
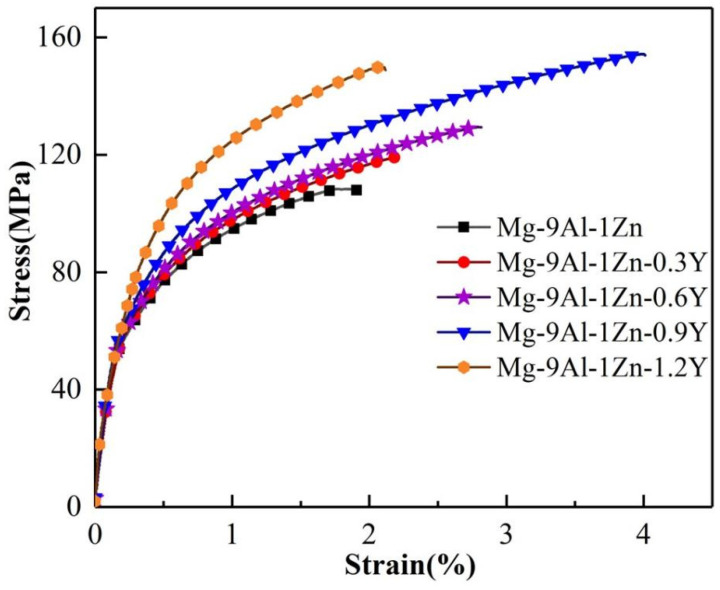
The stress–strain curve of the Mg-9Al-1Zn-xY alloy.

Here, we describe the mechanical properties of the Mg-9Al-1Zn alloy at room temperature after adding various levels of Y elements in Figure 9. The yield strength (YS), ultimate tensile strength (UTS), and elongation of the Mg-9Al-1Zn alloy without added Y element are 70 MPa, 108 MPa and 1.9%, respectively. When 0.3 wt.% content of element Y is added, the YS and UTS of the Mg-9Al-1Zn alloy increase to 75 MPa and 119 MPa, respectively. The mechanical properties of the Mg-9Al-1Zn alloy continuously improve as the amount of element Y increases. When the Y-element content increases to 0.9 wt.%, the YS, UTS and elongation of the Mg-9Al-1Zn alloy reach 87 MPa, 154 MPa and 4%, respectively, exhibiting improvements of 24%, 43%, and 110% compared to the Mg-9Al-1Zn alloy. The main reason for the improvement of the mechanical properties of the Mg-9Al-1Zn alloy is that the addition of the Y element realizes the refinement of the matrix grains and significantly reduces the size of the eutectic Mg_17_Al_12_ phase of the alloy, considerably strengthening the alloys with enhanced mechanical properties. In addition, due to the formation of a large number of Al_2_Y particles in the Mg-9Al-1Zn alloy, which is a highly stable phase, the alloy is able to effectively restrict the movement of dislocations, thus playing a role in dislocation strengthening. The grain refinement introduces a large number of grain boundaries, and the increase in grain boundaries can also effectively hinder the sliding of dislocations, further enhancing the mechanical properties of the alloy. However, when the content of Y element is increased to 1.2%, the YS of the alloy is further increased to 90 MPa, but the elongation of the alloy decreases significantly. This may be due to the fact that the addition of excessive Y elements increases the content of the Al_2_Y phase in the matrix and produces agglomeration phenomena, resulting in a greater stress concentration during the tensile process and ultimately leading to a reduction in the elongation of the alloy.

## 4. Conclusions

In summary, in this work, the influence of adding varying amounts of Y elements on the microstructure evolution of Mg-9Al-1Zn alloys was investigated in detail, and the grain refinement mechanism was characterized by OM, SEM and TEM analyses, and the effect of Y elements on the mechanical properties of Mg-9Al-Zn alloys was investigated. The main conclusions are as follows.

The grain size of Mg-9Al-1Zn alloy decreases with the increase in Y element content. Due to the excellent orientation relationship between the in situ formed Al_2_Y phase and α-Mg grains, the grain size of Mg-9Al-1Zn alloy was reduced from 334.2 μm to 134.6 μm when the added Y element content was 1.2 wt.%, and the efficiency of grain refinement increased by up to 60%.

It was observed by SEM that the eutectic Mg_17_Al_12_ phase of the Mg-9Al-1Zn alloy can also be markedly refined by the addition of the Y element, and its morphology gradually transformed from a continuous network-like structure to a discontinuous point-like structure distributed at the grain boundaries.

The addition of Y element can greatly enhance the mechanical properties of Mg-9Al-1Zn alloy. It was found that the YS and UTS of the Mg-9Al-1Zn alloy reached 87 MPa and 154 MPa with the addition of 0.9 wt.% of Y element—24% and 43% higher than the original alloy. The improvement in mechanical properties is mainly attributed to the significant grain refinement of the Mg-9Al-1Zn alloy. Simple alloying element additions have enabled significant grain refinement and improved the mechanical properties of Mg-Al alloys, expanding their potential applications in aerospace, automotive, and other engineering fields.

## Data Availability

The original contribution presented in the study are included in the article, and further inquiries can be directed to the corresponding author.
